# Neuroprediction and A.I. in Forensic Psychiatry and Criminal Justice: A Neurolaw Perspective

**DOI:** 10.3389/fpsyg.2020.00220

**Published:** 2020-03-17

**Authors:** Leda Tortora, Gerben Meynen, Johannes Bijlsma, Enrico Tronci, Stefano Ferracuti

**Affiliations:** ^1^Department of Human Neuroscience, Sapienza University of Rome, Rome, Italy; ^2^Willem Pompe Institute for Criminal Law and Criminology/Utrecht Centre for Accountability and Liability Law (UCALL), Utrecht University, Utrecht, Netherlands; ^3^Faculty of Humanities, Vrije Universiteit Amsterdam, Amsterdam, Netherlands; ^4^Department of Computer Science, Sapienza University of Rome, Rome, Italy

**Keywords:** neuroprediction, artificial intelligence, recidivism, forensic psychiatry, risk assessment, neurolaw

## Abstract

Advances in the use of neuroimaging in combination with A.I., and specifically the use of machine learning techniques, have led to the development of brain-reading technologies which, in the nearby future, could have many applications, such as lie detection, neuromarketing or brain-computer interfaces. Some of these could, in principle, also be used in forensic psychiatry. The application of these methods in forensic psychiatry could, for instance, be helpful to increase the accuracy of risk assessment and to identify possible interventions. This technique could be referred to as ‘A.I. neuroprediction,’ and involves identifying potential neurocognitive markers for the prediction of recidivism. However, the future implications of this technique and the role of neuroscience and A.I. in violence risk assessment remain to be established. In this paper, we review and analyze the literature concerning the use of brain-reading A.I. for neuroprediction of violence and rearrest to identify possibilities and challenges in the future use of these techniques in the fields of forensic psychiatry and criminal justice, considering legal implications and ethical issues. The analysis suggests that additional research is required on A.I. neuroprediction techniques, and there is still a great need to understand how they can be implemented in risk assessment in the field of forensic psychiatry. Besides the alluring potential of A.I. neuroprediction, we argue that its use in criminal justice and forensic psychiatry should be subjected to thorough harms/benefits analyses not only when these technologies will be fully available, but also while they are being researched and developed.

## Introduction

Risk assessment is a crucial component of the criminal justice system. In recent years, there has been a growing interest in the development of new tools and techniques to improve risk assessment in the field of forensic psychiatry and criminal justice (Monahan and Skeem, 2015). Currently, more than 200 violence risk assessment tools, often integrated clinical-actuarial instruments, have been developed to predict violent, antisocial, and sexual behavior (Singh et al., 2014), and their use seems to be vastly increasing in criminal justice settings (Conroy and Murrie, 2007). The central aim of these methods is to identify high-risk and low-risk offenders correctly. Depending on the jurisdiction, they are used to inform a range of medico-legal decisions, for instance regarding sentencing, parole, civil commitment, death penalty, disposition in juvenile courts, and discharge following findings of insanity (Conroy and Murrie, 2007). In recent years, A.I. (Artificial Intelligence) is being used to enhance the predictive accuracy of risk assessment.

The use of algorithmic risk assessment has grown along with the research in the field of neuroimaging, leading to the development of ‘brain-reading’ techniques that are, to some limited extent, able to decode mental states based on a person’s brain activity (Haynes and Rees, 2006), or to classify people in groups based on their brain structure and functionality (Koutsouleris et al., 2012). A possible forensic application of the technique is to identify dangerous offenders. The combination of A.I. and neuroimaging has led to the development of what can be called ‘A.I. neuroprediction,’ which is the use of structural or functional brain parameters coupled with machine learning methods to make clinical or behavioral predictions. Perhaps, in the near future, A.I. neuroprediction could be more generally used to predict the risk of recidivism in forensic psychiatry and criminal justice. However, application of such techniques raises legal and ethical issues.

The purpose of this paper is to identify possibilities and challenges regarding the possible future use of A.I. neuroprediction of violence and recidivism in the fields of forensic psychiatry and criminal justice, discussing legal implications and ethical issues. In the next section, we will discuss risk-assessment techniques. In the third section, we consider current ‘brain-reading’ techniques that use neuroimaging coupled with A.I. In the fourth section, we provide an overview of recent neuroprediction studies using neuroimaging data coupled with A.I. to predict recidivism. In the fifth section, we discuss technological limitations and pitfalls of predictive analysis. Finally, in the sixth section, we discuss the ethical and legal issues raised by the application of these techniques.

## Risk Assessment: The State of the Art

In the past two decades, in both the US and Europe, interest in and research on violence risk assessment tools have significantly increased, providing different approaches varying from strictly actuarial tools, based on regression, to algorithmic risk assessment, providing a probabilistic estimate of reoffending, to structured professional judgment (Hart, 1998; Douglas and Kropp, 2002). Initially, actuarial methods dominated the field, but their predictive value remained quite limited, if not disappointing (Fazel et al., 2012).

Risk variables associated with an increased likelihood of an individual acting violently or aggressively include criminogenic needs (individual characteristics that increase the risk of recidivism), demographics, socioeconomic status, and intelligence (Gendreau et al., 1996). Risk factors are typically divided into static factors, that are historical and do not change (e.g., criminal history, offense types, childhood abuse) and dynamic factors that are, in principle, changeable and therefore they provide the opportunity for intervention, modifying future risk (e.g., impulsivity, drug use, social support, job, compliance with treatment). Some dynamic factors are quite stable, while others are more “fluid.” Dynamic factors need to be measured multiple times, sometimes within short intervals.

At present, the results of risk assessment tools, however, are far from perfect, especially for long term prediction; current criminal risk assessment tools show poor to moderate accuracy, and a good balance between false positives and false negatives is an issue that should be considered, depending both on the social and political context and on the stage of the criminal justice process in which the tool is used (Douglas et al., 2017). Generally, when a risk assessment tool classifies an individual as low-risk, it is often correct. However, if the tool classifies someone as high risk, this is quite often incorrect, and almost more than half of individuals targeted as high-risk are incorrectly classified (Fazel et al., 2012). False positives (defendants are predicted to re-offend, but they do not) seem to be more common than false negatives (defendants are predicted not to re-offend, but they do) (Fazel et al., 2012).

The result is that many people may be or remain incarcerated, while they do not pose a danger to society. As Fazel et al. (2012) wrote: “One implication of these findings is that, even after 30 years of development, the view that violence, sexual, or criminal risk can be predicted in most cases is not evidence-based.” This diagnosis of the current state of affairs makes it important to look for ways to improve risk assessment in forensic psychiatry and criminal justice.

Algorithms hold the promise of performing more accurate predictions of criminal behavior than classic approaches, commonly derived from various forms of regression analyses (Berk and Hyatt, 2015). They can be used to provide measures of individualized risk for future violence and help to make decisions about prevention and treatment, in order to minimize risk factors and accentuating protective ones. Risk assessment tools that incorporate machine learning are already in use in pretrial risk evaluation, sentencing, and rehabilitation (Kehl et al., 2017), and are potentially very useful in judicial decision-making, to guide “decisions regarding bail, probation/parole, court-ordered treatment, and civil commitment” (Poldrack et al., 2018).

## A.I. and Neuroimaging

Rapid advances in brain imaging and the growing influence of A.I. technologies in many areas of society, from social networks to health care and police force policies (Berk et al., 2018), have led to interest in the potential use of brain imaging combined with A.I. to improve risk assessment and prediction of future violent behavior.

Over the past decade, there has been a significant development of non-invasive anatomical and functional neuroimaging technologies, yielding a lot of data, and statistical machine learning methods are instrumental for analyzing vast amounts of neural data with increasing precision (Lemm et al., 2011) and modeling high-dimensional datasets (Abraham et al., 2014). Applying statistical machine learning methods to neuroimaging data is referred to as multi-voxel pattern analysis (MVPA) (Ombao et al., 2017, pp164–169). These methods, unlike conventional univariate approaches that analyze only one location at a time, allow for the identification of spatial and temporal patterns in the data, differentiating between cognitive tasks or subject groups with higher sensitivity, jointly analyzing data from individual voxels within a region (Haynes and Rees, 2006).

Since the advent of MVPA methods, they have become a popular approach in the “neuroimaging of healthy and clinical populations; studies have shown that information present in neuroimaging data can be used to decode” – to some extent – “intentions and perceptual states, as well as discriminate between healthy and diseased brains” (Bray et al., 2009). MVPA has been applied to decode visual features like edge orientation (Kamitani and Tong, 2005), the intention to perform one task rather than another (Haynes et al., 2007), sequential stages of task preparation (Bode and Haynes, 2009), and lie detection (Davatzikos et al., 2005; Blitz, 2017, pp. 45–58). While conventional functional imaging studies compare brain activity during different experimental conditions to identify which brain regions are activated by particular tasks, application of MVPA for brain-reading uses “patterns of brain activity to perform a reverse inference and decide what subjects are looking at or thinking about” (Cox and Savoy, 2003; Bray et al., 2009).

These techniques can be considered ‘brain-reading’ or ‘mind-reading’ techniques; they combine statistical machine-learning methods with neuroimaging data to reveal information about the brain/mind. Brain-reading has often been studied in the domain of visual perception, where it aims to show how experiences are encoded in the brain. Researchers recently succeeded in training a deep neural network^1^ to perform visual image reconstruction from the brain (Shen et al., 2019), decode visual content of dreams (Horikawa et al., 2013), and decode what the brain is ‘seeing’ by using A.I. to analyse fMRI scans from subjects watching videos (Wen et al., 2017). Despite promising findings, these methods still show many limitations that make it unlikely that a ‘general mind-reading technique’ will appear in the very near future. Nonetheless, the first simple applications have begun to emerge, including brain-computer-interfaces, studies on lie-detection and approaches for prediction of consumer decisions in the field of neuromarketing (Haynes, 2012, pp. 29–40).

Apart from making inferences regarding the occurrence and nature of mental states (Haynes, 2012, pp. 29–40), another field of application of MVPA techniques is classification. For example, it has been reported that it is possible to predict disease onset by distinguishing individuals within a group based on brain activity or classifying individual people into groups based on the brain data identifying patterns of brain activity or structures (Koutsouleris et al., 2012). Treatment responders can be distinguished from non-responders, by extracting patterns of activity or structural abnormalities that are predictive of abnormal cognitive development and particularly relevant for prediction of clinical outcomes from neuroimaging data (Bray et al., 2009). Some models are applied to discriminate between clinical groups such as Alzheimer Disease patients and cognitively normal elderly individuals (Klöppel et al., 2008), Parkinson’s disease patients and healthy controls (Rubbert et al., 2019), schizophrenic patients and healthy controls (Kim et al., 2016), or to detect brain function disorders, such as Autism and attention deficit hyperactivity disorder (ADHD) (Heinsfeld et al., 2018; Sen et al., 2018) and to discriminate between levels of personality traits, for example psychopathy (Steele et al., 2015).

Interesting results have also been reported about prediction of addiction outcomes; machine learning classifiers were able to predict substance abuse treatment completion in a prison inmate population using event-related potentials (ERPs) (Steele et al., 2014; Fink et al., 2016) and functional network connectivity (FNC) analyses of fMRI data (Steele et al., 2018). Furthermore, it turned out to be possible to identify ‘neural fingerprints’ to predict cocaine abstinence during treatment using CPM, a recently developed machine learning approach (Yip et al., 2019).

## A.I. Neuroprediction of Recidivism

Behavioral traits can be correlated, sometimes strongly, with features of the human brain, and this raises new possibilities for predictive algorithms to be developed, allowing the prediction of dispositions of an individual. These methods are referred to as “neuroprediction,” that is the use of structural or functional brain variables to predict prognoses, treatment outcomes, and behavioral forecasts (Morse, 2015). Even though at present it may sound like science fiction, with the continuing development of non-invasive neuroimaging techniques coupled with the growth in the computational power of algorithms, A.I. neuroprediction of recidivism is likely to become available in the near future.

Although there is still need to collect biomarkers of the “criminal” brain, research in the field of neurocriminology has generally focused on the analysis of structural and functional neuromarkers of personality disorders whose main characteristic consists of persistent antisocial conduct, such as ASPD (De Brito et al., 2009) and psychopathy (Umbach et al., 2015), because they appear to be the most correlated to high rates of recidivism (Coppola, 2018). Research shows that these particular clinical populations share many traits, such as behavioral disinhibition or a lack of empathy, that are supposed to have common neurobiological bases (Coppola, 2018).

For example, abnormalities in limbic and paralimbic regions have been observed in individuals with psychopathic traits (Anderson and Kiehl, 2012) and impairments related to the prefrontal cortex are associated with disinhibition, emotional lability, and impulsivity (Chow, 2000; Yang and Raine, 2009).

Still, all such neurocriminological findings, obtained using conventional methods, do not enable us at this moment to make predictions of future risk. However, incorporating neurodata in A.I. prediction models appears to open up this possibility.

A first step toward A.I. prediction models using neuroimaging data is a study conducted by Aharoni et al. (2013), who used fMRI data to predict recidivism. The authors showed that activation in the dorsal anterior cingulate cortex (dACC), a brain region associated with impulse control and error processing, during a go/no-go task appeared to be associated with rearrest. The probability that offenders with relatively low anterior cingulate activity would be rearrested was approximately double compared to an offender with high activity in this region, keeping all the other risk factors constant. Low anterior cingulate activity, therefore, might be a potential neurocognitive biomarker for persistent criminal behavior (Aharoni et al., 2013).

Recently, a study by Kiehl et al. (2018) used machine learning coupled with neuroimaging to test whether brain age could help predict rearrest. Chronological young age is considered one of the key risk factors for recidivism. Young defendants are more likely to engage in risky behavior. Kiehl proposes that brain age is a better measure to account for individual differences than chronological age. The results of his study show that a predictive model involving neural measures of brain age performed better than previous models including only psychological and behavioral measures.

Even more recently, a study by Delfin et al. (2019) shows that improvements in recidivism prediction in forensic psychiatry might be possible by incorporating neuroimaging data into A.I. risk assessment models. The authors showed that the inclusion of resting-state regional cerebral blood flow (rCBF) measurements in an extended A.I. prediction model, containing neural measurements from eight brain regions, leads to an increase in predictive performance over traditional, empirical risk factors in a long-term follow-up of forensic psychiatric patients. Interestingly, they used ‘classical’ risk assessment *combined* with neuroimaging, which showed a better prediction in a forensic psychiatric population than the classical factors alone (Delfin et al., 2019).

In sum, preliminary findings in A.I. neuroprediction studies have produced some promising results. Still, the possible use of A.I. and ‘brain-reading’ in forensic populations raises several ethical and legal concerns, and the field of criminal justice should be cautious about their future use.

It is crucial to balance the preservation of offenders’ individual rights on the one hand and the enhancement of public safety on the other.

## Predictive Analysis: Technological Limitations and Pitfalls

Despite the opportunities previously discussed regarding the future possible use of A.I. neuroprediction techniques, several limitations should be considered; indeed, research about prediction tools and their successful application is still a challenging task (Poldrack et al., 2019).

This issue is well-known in the field of computational psychiatry, in which studies combining machine learning approaches and neuroimaging-based single subject prediction of brain disorders aim to classify patients with heterogeneous disorders (Arbabshirani et al., 2017; Bzdok and Meyer-Lindenberg, 2018). These studies, interestingly, reported varying degrees of accuracy (Neuhaus and Popescu, 2018), raising concerns about the methodology (Cearns et al., 2019). In fact, there is a need for best practices in predictive modeling (Poldrack et al., 2019); a problem of neuroprediction models is that, even though they can manage complex data such as brain imaging scans, they need best practices to ensure enough statistical power to test them (Varoquaux, 2018). Several issues deserve attention here.

First, application of neuroprediction techniques requires an inference from group-level to individual predictions (Hahn et al., 2017). Another challenge concerns validation of the results in a new group – different from the data set that was used to train the algorithm. The validity of prediction models is assessed by their ability to generalize; for most learning algorithms, the standard practice is to estimate the generalization performance through a process called ‘cross-validation’: the dataset is split into two sets, a training set, used to fit the model, and a test set (Hastie et al., 2009; Varoquaux, 2018), and subsets of the data are used to train and test the predictive performance of the model iteratively.

Notably, the use of cross-validation with small samples can lead to highly variable and inflated estimates of predictive accuracy (Luedtke et al., 2019; Poldrack et al., 2019). Training machine learning algorithms requires large amounts of data; using a limited sample size may cause so-called *overfitting*, in which the model fits perfectly to the specific data set used to train it, but fits poorly to new and unseen data (Hastie et al., 2009; Poldrack et al., 2019). There is still no agreement on the adequate size of the dataset (Cearns et al., 2019); Luedtke et al. (2019) recommend to perform prediction analyses with samples no smaller than several 100 observations. Acquiring many samples, however, is often difficult and costly, especially when neuroimaging data are involved (Arbabshirani et al., 2017).

## Ethical and Legal Challenges

Prediction of recidivism using A.I. neuroprediction techniques evokes ethical and legal concerns, but also new possibilities. In what follows, we discuss some central ethical and legal issues.

First, we are confronted with the issue of *bias*. Since the advent of algorithmic risk assessment, a lot of reports have documented the fact that they are “dangerously” biased. The most famous case of supposed A.I. prejudice was reported by ProPublica in May 2016. COMPAS, an algorithm widely used in the US to guide sentencing by predicting the likelihood of a criminal reoffending, turned out to be racially biased against black defendants, according to ProPublica, because they were more likely than white defendants to be incorrectly classified as high risk (“false positives”)^2^ (Angwin et al., 2016). More recently, COMPAS has also been depicted as a “sexist algorithm” because its algorithmic outcomes seem to systemically overclassify women in higher-risk groups (Hamilton, 2019). Similarly, Predpol, an algorithm designed to predict when and where crimes will take place, already in use in several US states, in 2016 – after an analysis of the Human Rights Data Analysis Group – was found to result in police *unfairly* targeting certain neighborhoods. Officers were repeatedly sent to areas of the city with a high proportion of people from racial minorities, regardless of the effective true crime rate in those areas (Ensign et al., 2018). Furthermore, facial recognition software, increasingly used in law enforcement, represents another potential source of both race and gender bias (Raji and Buolamwini, 2019*).* Another example concerns Amazon’s ‘Rekognition’ software, which is used by some police departments and other organizations. In 2018, the ACLU found that it incorrectly matched members of the Congress with people who had been charged with a crime, disproportionally misidentifying African-American and Latino members of Congress as the people in mug shots^3^. A recent study evaluating the accuracy of three commercial gender classifiers showed that they performed better in classifying male subjects than female subjects, and all of them performed worst on darker-skinned females (Buolamwini and Gebru, 2018). Moreover, recent studies show that, if left unchecked, word embeddings A.I. exhibit outdated gender stereotypes, such as “*doctors*” being male and “*receptionists*” being female (Bolukbasi et al., 2016).

These findings have led to a broader debate about the *fairness* of risk assessment using A.I. (Berk et al., 2018). Although algorithmic risk assessments can be perceived as a means of overcoming human bias, they could still reflect prejudice and institutionalized bias. A.I. is trained on data – for example, criminal files – that may themselves reflect biases on the part of police officers, prosecutors, or judges. Based on these data, the algorithm then “concludes” that groups with certain traits are more dangerous than others, while in fact, this is the result of biased data. This sometimes is referred to as “bias in-bias out.” The results of A.I. prediction, in other words, highly depend on the quality of the data used. One advantage of using neuroimaging data – instead of police files – might be that neuroimaging does not reflect human bias. A.I. looks for correlations between brain activity and recidivism. Therefore, A.I. neuroprediction may offer possibilities to *decrease* bias in risk assessment. However, also since neuroprediction may be incorporated in existing risk assessment tools (see the study by Delfin et al., 2019), bias will remain a problem as long as there is no solution to bias in algorithms in general.

Furthermore, we should keep in mind that risk assessment is “quintessentially discriminatory” (Binns, 2017), meaning that it is all about classifying subjects into groups of low or high-risk individuals based on group traits. Neuromarkers for recidivism will undoubtedly be more prevalent in certain groups than in others. Treating groups of people differently because of their “brain” raises difficult questions about what constitutes unjustified unequal treatment. This question, however, is not typical of A.I. neuroprediction, but is a central issue in risk assessment and fairness in general (Nadelhoffer et al., 2012, p. 95; Tonry, 2014). Classifying people into groups based on their brain scan, even if useful to prevent possible harms, could easily lead to stigmatization and discriminating effects for those considered “high risk” in other aspects of the individual’s life. It could become a sort of *modern phrenology*, by discriminating between people based on what their brain looks like. While certain institutional procedures could discriminate against those considered “high risk,” stigmatization could be a more social process that excludes certain individuals based on their risk profile; for instance, stigmatization may be a consequence of sex offenders’ registration (Tewksbury, 2005).

A second point concerns *privacy*. The neurodata and other data used to predict recidivism can clearly also be of interest for other purposes. For instance, for insurance companies, or when screening job applicants. Who should have access to these data, and under which conditions? Should insurance companies have access to them, and if not, should they be able to request such a procedure in order to assess the risk of a particular candidate client? Clearly, in this case, data protection – and possible access – is a fundamental issue, already highly debated in algorithms used in the era of big data. Obviously, there is also a parallel with the current debate on the nature of consent and the degree of control citizens have regarding health information in biobanks. The discussion of commercialization of genetic/health information and rights of control (“biorights”) are likely to intensify in the coming years (see also Caulfield and Murdoch, 2017).

A third, related point concerns the probability of a *negative ‘self-fulfilling prophecy*.’ This qualm comes from recent studies, showing that receiving genetic risk information can actually *influence* your behavior, physiology, and subjective experience and change your overall risk profile (Turnwald et al., 2019). Researchers from Stanford University found that when people were told of a genetic tendency for either obesity or lower exercise capacity, acquiring this information had a physiological impact on their bodies, modifying how they responded to a meal or to exercise. A persistent discovery was that perceptions of risk altered health outcomes, therefore those informed of having the high-risk gene had a worse outcome than those informed of having the protective one (Turnwald et al., 2019). Following these findings, one may wonder how the mindset of people may be affected when you inform them about their own risk information, either genetic or neural, and how this could actually alter their risk profile. This shows that providing information may also require ethical and/or legal research and regulation.

Furthermore, it is still not clear how to exactly classify and conceptualize neurodata as risk factors. For example, in a study by Kiehl et al. (2018), a measure of brain age (gray matter) is used to predict recidivism. Chronological age is often considered a static factor, but when referring to brain measures, we should reflect on how they should be conceptualized among risk factors. For instance, given the plasticity of the brain, should we consider brain age as a dynamic or static risk variable? How do we evaluate an offender if, for example, brain age and normal age differs, and how would this modify his/her neuroprediction profile? If we consider neurodata as dynamic factors, and, as such, available to be modified through interventions, we could talk, instead of in terms of a pure “prediction,” in terms of targets for treatment and other intervention types. Used in this way, neuroprediction could help to prevent crime through more individualized correctional and socio-rehabilitative measures, and could also enable offenders to return to the community sooner. As in “personalized medicine” – a therapeutic approach in which an individual’s genetic and epigenetic information is used to tailor drug therapy or preventive care^4^ – neuroprediction could help to target interventions to the individual’s “needs.”

There is another effect of the emphasis on prediction that is relevant here. Currently, A.I. is used in the criminal justice system, mainly to predict recidivism. A.I. risk assessment typically does not offer a causal model of crime and therefore, is not designed to show opportunities to intervene and to mitigate risk (Berk, 2019, pp. 17–18). Barabas et al. (2018) conclude: “when risk assessments are used primarily as a predictive technology, they fuel harmful trends toward mass incarceration and growing inequality in the justice system.”

We should acknowledge that A.I. neuroprediction in the first place merely establishes correlations between brain images and the risk of recidivism. However, if it is indeed possible to develop interventions based on neurodata, this might offer offenders an opportunity to avoid incarceration (Nadelhoffer et al., 2012, pp. 85–86). This could be possible because, different from historical data and other risk variables, like a person’s demographic characteristics such as ethnicity, age, and gender, that cannot be changed, neurodata hold the potential to become targets for new rehabilitative interventions and prevention programs, aiming to reduce exposure to risk factors for psychopathic traits and preventing at-risk individuals from engaging in criminal behavior later in life (Ling and Raine, 2018).

This is particularly important since the prison environment may have negative effects on neurocognitive functioning. In fact, studies found that incarceration might lead to reduced self-control (Meijers et al., 2018). Still, the possibility of intervention also entails its own ethical and legal issues: for an offender, it may be hard to choose between a deprivation of liberty and undergoing (possibly somewhat invasive) treatment, especially in light of the right to refuse medical treatment (Meynen, 2018). However, this again is not a problem that is typical of interventions based on “A.I. neuroprediction.”

A fourth, and related, issue concerns *consent and coercion*; if and when these techniques will be fully developed and are ready to be used, there may be a possibility of performing cognitive liberty violations forcing people to undergo scans without consent for sentencing or punitive purposes (Ligthart, 2019; Meynen, 2019). Coercion, both technical and ethical or legal, not only relates to the force used, because not all the imaging techniques allow for this, but also to their use within the context of a threat or an offer that cannot be refused (Meynen, 2017). One way to counter this issue is to strictly regulate informed consent for neuroprediction tests.

Fifth, we should take into account something called the “seductive allure” that neuroimaging exerts on courts. Juries and judges apparently tend to overestimate the accuracy of neuroscientific evidence, and, although neuroimaging aims to reduce uncertainty and to increase the objectivity in forensic settings, the use of neuroimaging in courts is at risk of being misleading, due to cognitive biases in the evaluation of evidence (Scarpazza et al., 2018). Introducing neuroprediction could therefore lead to some overreliance on neurodata.

Furthermore, machine learning algorithms are considered to be ‘*black-boxes of decision-making’*; the way in which they perform decisions is not fully comprehensible to stakeholders, and not even to expert data scientists (London, 2019; Pedreschi et al., 2019). In addition, we have to be cautious about what is called the “the control problem”; i.e., the tendency of human operators to become complacent with machines, devolving responsibility and becoming over-reliant on the outputs of autonomous systems, even when they are biased (Pedreschi et al., 2019). In order to avoid overreliance, it seems important for A.I. systems to be transparent: it should be possible to explain to judges and a jury how they produce their results (Gunning and Aha, 2019), and stakeholders should be capable to appropriately trust and manage these tools, reasoning on how a specific output is given and on the basis of what rationale (Pedreschi et al., 2019). Even if this is actually complicated by the fact that most risk assessment algorithms are proprietary, it seems important for society that A.I. algorithms can be made intelligible, in order to be accountable for their decisions (Weld and Bansal, 2019).

Of note, legal systems may have criteria for the admissibility of scientific evidence in the courtroom. For instance, in the US legal context *Daubert* and *Frye* are used as standards. As we do not focus on specific legal systems, we will not go into this in more detail, but clearly such legal criteria would be relevant for courtroom use of new technologies (Shats et al., 2016).

Moreover, it is important to make a decision about the required accuracy of these technologies. Current risk assessment tools often have an AUC of about 0.70 (Douglas et al., 2017); is that enough for such algorithms, or should the threshold be higher, like 0.80 or 0.90? These are normative choices that have to be made before deciding to allow the use of this kind of technology to prevent crime.

Additionally, we need to consider the lack, at present, of a ‘true’ prediction model; a limitation of the papers previously discussed is that, instead of talking about ‘pure’ prediction, they can be classified as *postdiction* studies; postdiction generally relates to retrospectively making an assertion or deduction about an event based on information available after the event (Yamada et al., 2015) but, as applied to the context of statistical models, the distinction between prediction and postdiction is about whether the assessment of the model’s success involves the same data as were used to build the model or new data not used in model construction (Gauch and Zobel, 1988; Hastie et al., 2009). Research suggests that models for predictive applications, such as biomarkers, require larger sample sizes than standard statistical approaches (Varoquaux, 2018). Furthermore, in the studies discussed before, data about neuromarkers of recidivism have been collected after the commission of crimes, so we cannot establish when brain differences observed developed (Cope et al., 2014). A future challenge is to develop a true prediction model, able to identify those at the highest risk for committing crimes, and research in neuroimaging coupled with A.I. may be the key in developing such model.

Finally, there appears to be a more remote problem, looming on the horizon. Suppose that these A.I. algorithms – either with or without brain imaging – become really good predictors, wouldn’t that introduce a form of determinism we have not witnessed before? The A.I. system may be considered to have some “divine” foreknowledge about what will happen, which may have negative effects on the freedom people experience and exert. A belief in free will seems to have positive effects (Crescioni et al., 2016; Feldman et al., 2016).

Still, the more pressing concern nowadays is that we are not quite good at predicting risk – even with A.I. – and that we nonetheless often apply sanctions based on the supposed dangerousness of the offender. If A.I. becomes more accurate with the help of neuroimaging, it could reduce the number of persons incorrectly classified as high risk and can therefore reduce sanctions that in fact are not legitimate, helping to interrupt the so-called “cycles of crime” (Barabas et al., 2018).

## Conclusion

There is still a way to go before combined neuroscience and AI-based violence risk assessment tools can be implemented in the criminal justice system. Still, A.I. is already being used in criminal justice systems. Because of the far-reaching consequences of these type of technologies – and also given some rapid developments in recent years – it is important to consider ethical and legal concerns. Besides discussing technological limitations and pitfalls of predictive analysis, we identified six key issues deserving attention: dealing with bias, privacy, the possibility of a ‘self-fulfilling prophecy,’ coercion and consent, the allure of neuroimaging data and the need for A.I. systems to be explainable. Finally, we pointed to the more remote issue of how highly accurate predictions might introduce a form of determinism we have not witnessed before – but this is still far away.

Still, we would like to emphasize that accurate risk prediction is extremely valuable for both safety and justice reasons. Therefore, in principle, we argue that technologies that may be helpful in this respect should at least be explored, and if ready, used in criminal justice and forensic psychiatry. In addition, neuroprediction and A.I. bring their own, in a way new, ethical and legal challenges, and we will have to deal with them – preferably before the technologies are used. More specifically, we have to find solutions to prevent systems from reflecting our own human biases in order to enable them to provide objective and trustworthy data.

Therefore, we argue that the use of AI-based systems in criminal justice and forensic psychiatry should be subjected to substantial regulation to protect citizens from system errors or misuse. On such basis, we highlight the importance of accurate harms/benefits analyses not only when these technologies will be fully available, but also while they are being researched and developed.

## Author Contributions

LT, GM, and SF conceived the content of the manuscript and wrote and revised the manuscript. LT drafted the manuscript. JB and ET wrote and revised the manuscript. All authors read and approved the final manuscript.

## Conflict of Interest

The authors declare that the research was conducted in the absence of any commercial or financial relationships that could be construed as a potential conflict of interest.

## Footnotes

1 A neural network is “a system composed of many simple processing elements operating in parallel whose function is determined by network structure, connection strengths, and the processing performed at computing elements or nodes.” [DARPA Neural Network Study (U.S.)., United States. Air Force. Systems Command., Lincoln Laboratory. (1989). DARPA neural *network study final report.* Lexington, Mass.: The Laboratory].

2 The company that produced the Compass algorithm, Northpointe, claimed in a report that the accuracy in the prediction of violence for both groups of defendants was the same: around 70% of crimes were predicted correctly (see Dieterich et al., 2016, COMPAS risk scales: demonstrating accuracy equity and predictive parity. Retrieved from www.documentcloud.org/documents/2998391-ProPublica-Commentary-Final-070616.html). The different levels of false positives among black defendants and white defendants were to be attributed, according to Northpointe, to different base rates in the prevalence of crime among black and white defendants. It is possible to have the algorithm acquire the same level of false positives over groups with a different base rate. However, this comes at the cost of reduced accuracy. There is an extensive literature on fairness in A.I. prediction, and its trade-offs (Berk et al., 2018). The text about these algorithms is partially based on Cossins (2018).

3 https://www.aclu.org/blog/privacy-technology/surveillance-technologies/amazons-face-recognition-falsely-matched-28

4 https://www.nature.com/subjects/personalized-medicine
